# Keratin–PNIPAM Hybrid Microgels: Preparation, Morphology and Swelling Properties

**DOI:** 10.3390/gels10060411

**Published:** 2024-06-20

**Authors:** Elena Buratti, Maddalena Sguizzato, Giovanna Sotgiu, Roberto Zamboni, Monica Bertoldo

**Affiliations:** 1 Department of Chemical, Pharmaceutical and Agricultural Sciences, University of Ferrara, Via L. Borsari 46, 44121 Ferrara, Italy; maddalena.sguizzato@unife.it (M.S.); monica.bertoldo@unife.it (M.B.); 2 Institute for Organic Synthesis and Photoreactivity (ISOF), National Research Council, Via Gobetti 101, 40129 Bologna, Italy; giovanna.sotgiu@isof.cnr.it (G.S.); roberto.zamboni@isof.cnr.it (R.Z.)

**Keywords:** hybrid, microgel, PNIPAM, keratin, swelling, morphology

## Abstract

Combinations of synthetic polymers, such as poly(N-isopropylacrylamide) (PNIPAM), with natural biomolecules, such as keratin, show potential in the field of biomedicine, since these hybrids merge the thermoresponsive properties of PNIPAM with the bioactive characteristics of keratin. This synergy aims to produce hybrids that can respond to environmental stimuli while maintaining biocompatibility and functionality, making them suitable for various medical and biotechnological uses. In this study, we exploit keratin derived from wool waste in the textile industry, extracted via sulfitolysis, to synthesize hybrids with PNIPAM microgel. Utilizing two distinct methods—polymerization of NIPAM with keratin (HYB-P) and mixing preformed PNIPAM microgels with keratin (HYB-M)—resulted in hybrids with 20% and 25% keratin content, respectively. Dynamic light scattering (DLS) and transmission electron microscopic (TEM) analyses indicated the formation of colloidal systems with particle sizes of around 110 nm for HYB-P and 518 nm for HYB-M. The presence of keratin in both systems, 20% and 25%, respectively, was confirmed by spectroscopic (FTIR and NMR) and elemental analyses. Distinct structural differences were observed between HYB-P and HYB-M, suggesting a graft copolymer configuration for the former hybrid and a complexation for the latter one. Furthermore, these hybrids demonstrated temperature responsiveness akin to PNIPAM microgels and pH responsiveness, underscoring their potential for diverse biomedical applications.

## 1. Introduction

Hybrid systems are materials in which different components are blended at the nanometer or molecular level that can synergistically combine the advantageous properties of the constituents, creating new physico-chemical properties and leading to enhanced performance [1,2,3]. They find application across diverse fields, including optics, electronics, soft robotics, mechanics, catalysis, sensors, environmental remediation, energy conversion and storage, and biomedical applications [1,2,3]. Hybrid materials are usually obtained from combinations of molecules of different natures, such as inorganic–organic and synthetic–natural components, or by combinations at different scales, such as macro–micro-/nanostructures [4].

Among these materials, hybrid systems combining synthetic polymers with natural biomolecules have emerged as promising candidates in the biomedical field, offering tailored properties and enhanced functionalities [5,6,7,8,9,10]. One of the most relevant examples is the combination of proteins with synthetic polymers [11,12], which has been demonstrated to affect cell adhesion, differentiation and proliferation and is thus appealing for applications such as drug delivery, tissue engineering and wound healing [5,13,14,15,16]. In this context, the integration of poly(N-isopropylacrylamide) (PNIPAM) microgels with keratin, a fibrous protein, offers a promising approach for the development of novel biomaterials with a wide range of applications. Indeed, a limitation of poly(N-isopropylacrylamide) on its own is its insufficient bioactivity, which restricts its effective interaction with biological systems. Conversely, keratin by itself may lack the necessary physical properties or functionalities needed for certain applications, such as thermoresponsiveness. Hybridization mitigates these limitations by integrating the distinct advantages of both materials.

Poly(N-isopropylacrylamide) is a thermoresponsive polymer known for exhibiting a lower critical solution temperature (LCST) behavior at approximately 32 °C in aqueous solutions [17]. At this temperature, the polymer undergoes a conformational transition: below the LCST, the chains are hydrated and dispersed in water, while above it, they become insoluble, resulting in a sharp yet reversible coil-to-globule transition. This transition temperature is very appealing, especially in biomedical applications, since it is close to the human body temperature. Microgels are colloidal particles in the range of nano- or micrometers composed of a three-dimensional crosslinked network dispersed in a suitable solvent. Compared to macroscopic hydrogels, microgels have a larger surface area-to-volume ratio, which makes them exhibit faster responsiveness to environmental changes (e.g., pH and temperature). This characteristic, along with their easy injectability, makes microgels appealing for medical applications such as drug delivery and minimally invasive procedures [18]. When PNIPAM is polymerized as a microgel, the analogue of LCST demixing observed in linear chains is represented by the occurrence of a so-called volume phase transition (VPT), where the microparticles change from a swollen and hydrated state to a collapsed and dehydrated one [19]. The transition allows tuning of the size of the particles, the hydration state and the swelling behavior, which is a very appealing feature for a variety of nano- and biotechnological applications [20,21,22]. Notably, in the biomedical field, the particles are exploited for controlled drug release, biosensing and building responsive surfaces for cell culturing [23,24,25,26,27,28,29,30,31,32,33]. For these applications, the impact of particle structure—specifically, size, crosslinker content, presence of co-monomers, etc.—on microgel performance has been extensively studied [29,31,34,35,36,37]. Achieving the desired outcomes is relatively straightforward by adjusting the synthesis parameters.

Unfortunately, the same control is not so easily achievable with naturally occurring biopolymers, though, as has been mentioned, they can guarantee high availability, biocompatibility, biodegradability and bioactivity [38,39,40]. Proteins are of particular interest because of their diverse structures, which contain various amino acid chains, providing many functional groups compared to other biopolymers, such as lipids or carbohydrates. In this work, we exploited keratin, one of the most abundant proteins and the major component of hair, feathers, wool, nails and horns of mammals and birds [41]. Keratin represents a family of structural proteins, classified as hard keratin or soft keratin, depending on the source—hair/nails or epithelial tissues, respectively [42]. The polypeptide chain of keratin is rich in cysteine (7–20 wt.% of the total amino acids) [43,44], an amino acid containing a sulfhydryl group (SH), capable of forming disulfide bridges between chains, which give the protein a solid, stable tertiary structure that is sparingly soluble in water [45,46]. Due to this characteristic, the management of keratin-containing wastes, which amount to millions of tons annually [47], poses significant challenges. Common disposal methods, including burial, open dumping, burning, landfilling, composting, mechanical grinding and incineration, often result in substantial environmental harm [47]. For this reason, new technologies have recently been developed to recover and transform keratin waste into new materials [48,49] with innovative properties, such as acid or alkaline hydrolysis, reductive extraction, oxidative sulfitolysis, ionic liquid dissolution, enzymatic hydrolysis, hydrothermal treatment, and steam explosion [48]. After these treatments, keratin is usually water-soluble and thus exploitable to create new materials for biomedical applications, since it exhibits intrinsic capabilities to enhance cell adhesion, proliferation and tissue regeneration [48,50]. The keratin employed in this study derived from wool waste in the textile industry and was extracted via sulfitolysis. It was demonstrated that this method facilitated the production of high-molecular-weight macromolecules, as it selectively cleaved disulfide bonds while preserving amino acid linkages [51,52].

Recently, various keratin-based systems have been developed for biomedical applications, primarily in tissue engineering, in the form of hydrogels [53,54,55,56,57], nanofibers [58,59] and films [60,61,62,63]. In these applications, keratin has been used either alone [55] or in combination with other polymers, such as PNIPAM [54,64,65,66,67], gluten [68], polyamide 6 (PA6) [52], poly(vinyl alcohol) (PVA) [57], polylactic acid (PLA) [69] and poly(ethylene glycol) (PEG) [70]. Regarding keratin/PNIPAM hybrids, it was observed that keratin did not affect the critical temperature of the hydrogels. However, the amount of biopolymer influenced the degree of hydrogel contraction, as well as the behavior at different pH levels and the mechanical strength [54]. Moreover, it was proven that grafting PNIPAM on keratin improved the thermal stability of the hybrid compared with the original keratin [66]. Additionally, the loading and release of drug molecules were tested, exhibiting response to temperature and pH [65], confirming the potential use of these hybrids as drug carriers [67].

However, while the described materials exist at the macroscopic scale (hydrogels), keratin-based systems at the micro- or nanoscale (microgels and nanogels) have not yet been prepared. Numerous examples of PNIPAM microgels combined with other biopolymers have been documented. Mainly, polysaccharides have been used, obtaining hybrids such as PNIPAM/dextran particles [71], (starch nanoparticle)-*co*-PNIPAM microgels [72], pullulan-based nanogels grafted with PNIPAM [73] and PNIPAM–chitosan copolymer microgels [37,74,75]. However, only a few examples can be found at the microscale in combination with keratin; for instance, the protein was grafted with PNIPAM brushes [66] to create nanoparticles with the biopolymer inside the core.

In our work, we prepared, for the first time, hybrids composed of PNIPAM and keratin in the form of microgels. The hybrids were synthesized using two different methods: in one approach, the microgel was obtained by polymerizing NIPAM in the presence of keratin (HYB-P); in the other, a preformed PNIPAM microgel was mixed with keratin, resulting in a physical mixture of the components (HYB-M). The composition and morphology of the two hybrids were analyzed using spectroscopic techniques (FTIR and NMR) and microscopic analysis (TEM), respectively, and compared to those of the pure components, PNIPAM and keratin. Additionally, the swelling behavior of the particles was evaluated as a function of temperature and pH using dynamic light scattering (DLS).

## 2. Results and Discussion

### 2.1. Synthesis of Hybrid PNIPAM/KER Microgels

**Figure 1:**
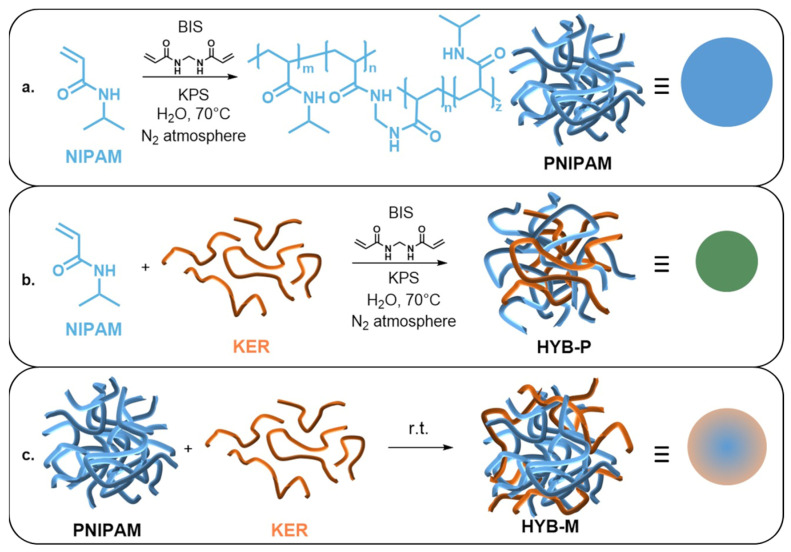
Schemes of the synthesis of (**a**) pure PNIPAM, (**b**) PNIPAM–keratin hybrid microgel by polymerization of NIPAM in presence of keratin (KER) and (**c**) PNIPAM–keratin hybrid microgel by mixing a preformed PNIPAM microgel with keratin (KER).

**Figure 2:**
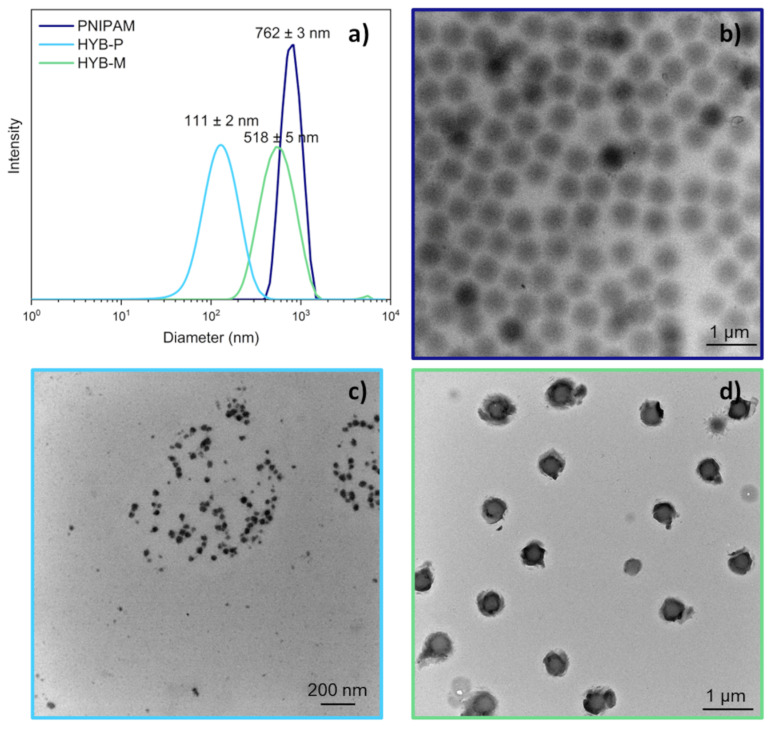
Microgels’ size distribution as determined by DLS analysis (**a**) and representative TEM micrographs of PNIPAM (**b**) (8500× mag), HYP-P (**c**) (28,000× mag) and HYB-M (**d**) (8500× mag) microgels.

The polymerization of NIPAM in water at 70 °C, in the presence of 25 wt.% keratin (KER) and BIS as a crosslinker (Figure 1), resulted in the formation of colloidal microgel particles (HYB-P) with an average diameter of approximately 110 nm at 20 °C, as determined by DLS analysis (Figure 2a).

The same polymerization carried out in the absence of keratin (Figure 1a) resulted in much larger colloidal particles (PNIPAM), approximately 760 nm in diameter, as determined by DLS analysis (Figure 2a). TEM analysis confirmed the colloidal nature of both samples, revealing a size of <100 nm for particles obtained in the presence of keratin (Figure 2d) and approximately 550 nm in its absence (Figure 2b). The values obtained by the two methods were reasonably consistent, considering the dehydration of particles onto the TEM grid.

Adding keratin to PNIPAM microgels to achieve a composition of PNIPAM/KER 75/25 by weight (Figure 1c) resulted in a new hybrid sample (HYB-M) with particles slightly smaller than those of PNIPAM but larger than those in HYB-P, as observed by TEM analysis (Figure 2c). These particles exhibited less smooth edges and a darker corona compared to pristine PNIPAM. Keratin, being rich in sulfur—a heavier atom than the carbon, nitrogen and hydrogen found in PNIPAM—scatters the electron beam more prominently, resulting in darker areas under TEM observation. Therefore, the dark corona of particles was primarily attributed to keratin. No other material was detected on the TEM grid, suggesting complete adsorption of the added protein onto the PNIPAM particles. DLS analysis of HYB-M supported this observation, as the scattered light could be well fitted by a single peak, corresponding to an average size of approximately 520 nm. Additionally, DLS analysis of the keratin solution revealed particles of 240 nm (Table 1), which were not detected in HYB-M. Despite protein adsorption, the particle size was smaller in HYB-M than in pristine PNIPAM. This reduction was attributed to shrinking due to adsorption, although an effect due to a change in the refractive index of the particles cannot be excluded.

Particle size reduction was also observed when PNIPAM microgels were prepared in the presence of chitosan [75]. The authors attributed the size reduction to the surfactant role of chitosan via electrostatic interaction between negatively charged particles and positively charged chitosan. Here, we observed a comparable effect when the protein was present during synthesis or added afterward. Furthermore, unlike in the case of chitosan, no interaction between oppositely charged species can be invoked. Therefore, two distinct effects likely influence particle size: (a) the surfactant role of the protein during synthesis, acting as a PNIPAM stabilizer through hydrophobic interactions or the formation of covalent bonds, and (b) reduction in the microgels’ swelling ability by complexation with the protein and a decrease in the sites available for hydrogen bond formation with water.

In HYB-P, the sulfur content determined by elemental analysis was 0.99%—slightly lower than the expected value of approximately 1.3% based on the keratin content (Table 1). However, in HYB-M, a good agreement was observed between the expected and observed sulfur content. The keratin used in this study has been reported to include a fraction with a molecular weight smaller than 6 kDa [51]. After dialysis using the same membrane employed for the purification of HYB-P, keratin lost 23% of its weight. Assuming a comparable loss for the keratin component in HYB-P, the sample composition would be approximately 80% PNIPAM and 20% KER. The corresponding expected sulfur content would be 1.04%, in good agreement with the observed sulfur content determined by elemental analysis (Table 1).

**Figure 3:**
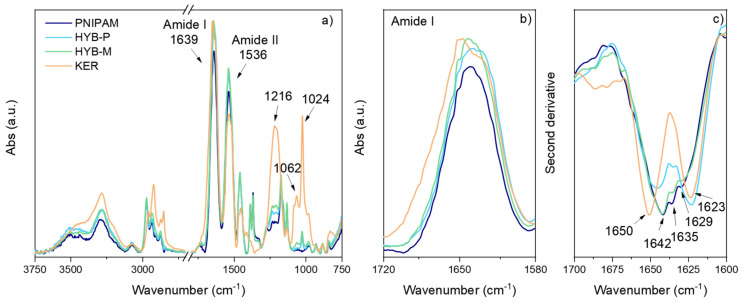
Comparison of the ATR spectra of PNIPAM, HYB-P, HYB-M and KER (**a**). Detail of the amide I spectral region (**b**). Second derivative of the amide I spectral region (**c**). Individual spectra are reported in the Supplementary Materials (Figures S1–S4).

The Ellman assay indicated that only 0.42% by mol of keratin residues contain -SH groups consisting of cysteine, suggesting that most of the detected sulfur should belong to oxidized or S-S cysteine residues. Accordingly, in the infrared spectrum of keratin, an intense band was detected at 1024 cm^−1^ due to the presence of the RSSO_3_^−^Na^+^ salts, as a consequence of the sulfitolysis process, while the S-H stretching expected at 2550 cm^−1^ was not detected (Figure 3a). The amount of -SH in HYB-P corresponded to 1.00% by mol of the protein residue, which is more than double the value detected in pristine keratin. This difference is attributed to the scarce amount of -SH in the keratin fraction lost by dialysis, which instead contains oxidized cysteine residues.

The stretching bands of C-S linkages at 1024 cm^−1^ and 1062 cm^−1^ were also detectable in the spectra of HYB-P and HYB-M (Figure 3a), supporting the presence of keratin in HYB-P. Further support was provided by the presence in the infrared spectra of hybrids of a band with a maximum at 1216 cm^−1^ superimposed on some weak adsorption of PNIPAM. This band in keratin is very strong and is due to the so-called amide III band [69]. Other relevant bands of keratin fall in the 1700–1600 cm^−1^ (amide I) and 1600–1500 cm^−1^ (amide II) ranges [76,77]. Amide I absorption primarily arises from C=O stretching vibration, while amide II derives mainly from in-plane N−H bending (40−60% of the potential energy) with minor contributions of C−N (18−40%) and C−C (about 10%) stretching vibrations. PNIPAM also presents amide I and amide II vibration bands in its infrared spectrum due to the presence of amide groups in the side chains. The maximum adsorptions are at 1639 and 1536 cm^−1^, for amide I and amide II, respectively (Figure 3).

### 2.2. Structural Characterization of the Hybrid Microgels

**Figure 4:**
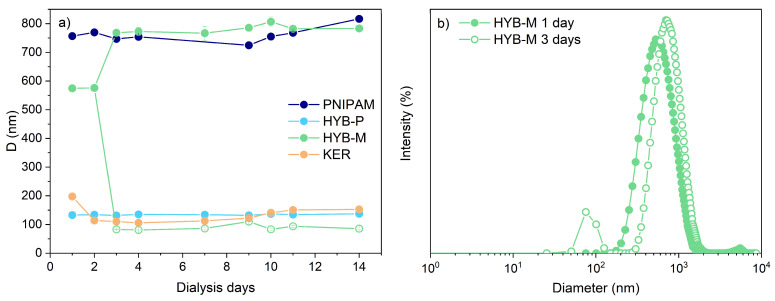
(**a**) Particle diameters, determined by DLS analysis at 25 °C, of HYB-P, HYB-M, KER and PNIPAM during dialysis into a membrane with a cutoff of 100 kDa for two weeks. (**b**) Particle size distribution, analyzed by DLS, of HYB-M after 1 day and 3 days of dialysis.

To shed light on the possible presence of covalent bonds between keratin and PNIPAM chains in HYB-P, the sample was dialyzed using a very high cutoff membrane (100 kDa) for two weeks. HYB-M, keratin and PNIPAM were utilized as references. Throughout the dialysis process, small aliquots of the samples were withdrawn from the membranes periodically and analyzed using dynamic light scattering (DLS) (Figure 4). Interestingly, the particle diameters remained relatively unchanged over the dialysis period for all samples, except for HYB-M. In the case of HYB-M, after the third day of monitoring, two distinct peaks were observed via DLS analysis instead of a single peak. One peak was larger than the initial value and corresponded to the diameter of PNIPAM, while the second peak was smaller and similar to the diameter registered for the dialyzed keratin (Figure 4). HYB-M was prepared by simply mixing preformed PNIPAM microgels and keratin, so it was not surprising that some keratin diffused out of the membrane, leading to the separation of the two components. However, it was unexpected to observe that some keratin did not exit from the membrane, even after two weeks. This observation may suggest the presence of chains extended through stable disulfide bridges that are water-soluble due to their nearly linear or only branched structure.

No significant change in particle size or volume within the membrane was observed for HYB-P or PNIPAM during the dialysis period (Figure 4a). Conversely, the volume increased for HYB-M and KER due to water–keratin exchange. These findings support the formation of a graft copolymer structure (keratin-g-PNIPAM) in HYB-P.

Zhu et al. [64] also reported the formation of keratin-g-PNIPAM copolymers through radical polymerization of NIPAM in the presence of keratin. However, they observed a decrease in SH groups on keratin after the process, suggesting the involvement of thiols as transfer agents during radical polymerization. In our case, the SH group quantity did not decrease post-polymerization, making a similar grafting mechanism unlikely. Nevertheless, radical transfer to other functional groups on keratin remains possible. Grafting onto and from polypeptides with low SH group contents during free radical polymerization of acrylic and methacrylic monomers has been frequently reported. Persulfate, in particular, has been effective as an initiator in promoting the formation of graft copolymers from gelatin [78,79,80,81,82,83,84,85], casein [86], silk [87,88], etc. The most commonly proposed mechanism involves the abstraction of hydrogen radicals from functional groups present on the protein (e.g., -COOH, -NH2, -OH and -SH) by the initiator or radicals on the growing polyacrylic chains. The resulting radical on the protein backbone may serve as an initiation site for new acrylic chains, which then grow from the protein. The presence of crosslinkers further enhances the likelihood of grafted polymer chain formation [85].

**Figure 5:**
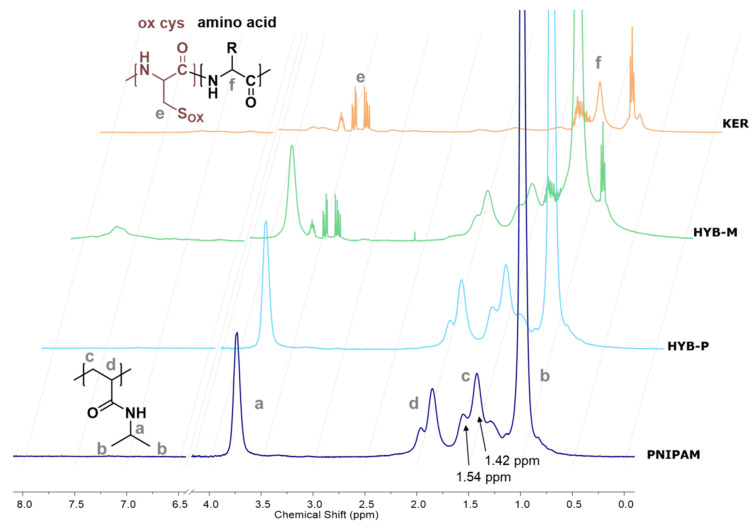
Comparison of the ^1^H NMR spectra of keratin (KER), PNIPAM, HYB-P and HYB-M in D_2_O. In the PNIPAM spectrum, the polymer structure with labels (a–d) for the assigned peaks are reported. In the keratin spectrum, the structure of the macromolecular chain is reported with labels (e and f), showing the oxidized cysteine (ox cys) repeating unit and a generic amino acid repeating unit. In the oxidized cysteine structure, S_OX_ can be SOH (sulfenic acid), SOOH (sulfinic acid) or SOOOH (cysteic acid). Individual spectra are reported in the Supplementary Materials (Figures S5–S8).

The ^1^H NMR spectrum of HYB-P, compared with the spectra of PNIPAM and keratin, only showed signals attributable to PNIPAM (Figure 5). In contrast, HYB-M exhibited signals from both PNIPAM and keratin. As the presence of keratin in both samples was confirmed by sulfur in the elemental analysis data and infrared spectroscopy, the discrepancy observed in NMR signals may be attributed to differences in keratin mobility in the two samples. Specifically, ^1^H NMR can distinguish between highly mobile (liquid-like) and immobile (solid-like) protons based on magnetic relaxation times [89]. Protons from species unable to undergo isotropic reorientation fast enough to average out intermolecular and intramolecular dipole–dipole interactions exhibit strongly dipolar broadening and may be invisible in conventional high-resolution NMR experiments [90]. The complete absence of keratin signals in the HYB-P spectrum may indicate strong coupling and/or entanglement of keratin with PNIPAM chains in the sample, preventing its rearrangement within the experimental time scale.

PNIPAM microgels synthesized under standard batch conditions typically exhibit a denser core with dangling chains in the outer sphere [91,92,93]. In HYM-M, keratin has been observed to predominantly adsorb onto the outer sphere of microgels, where it is more likely to rearrange freely, making its signals detectable under standard NMR experiments in the liquid phase. Conversely, in HYB-P, keratin was present during microgel synthesis and may have been trapped within the microgel core.

A detailed analysis of the amide I absorption bands in the infrared spectra mentioned above (Figure 3) provides insights into the protein’s secondary structure. Amide I is frequently utilized to study the conformational arrangements of proteins, including keratin [76,77,94,95,96]. The band maximum near 1655 cm^−1^ typically corresponds to α-helices [76], which shifts downwards with increasing helix length and in H_2_O. β-sheets exhibit a strong band near 1630 cm^−1^ [76] and a weaker band near 1685 cm^−1^. Unfolded proteins typically display a broad, featureless amide I band centered around 1650 cm^−1^, characteristic of unordered structures.

Band positions were accurately determined using the second derivative method [97]. For pure keratin (KER), two main minima at 1623 cm^−1^ and 1650 cm^−1^ were observed (Figure 3b), corresponding to β-sheets and α-helix structures, respectively. Other less intense signals were also present at 1670 cm^−1^, 1678 cm^−1^ and 1686 cm^−1^, attributed to β-turns and various configurations of β-sheets [97].

In the case of PNIPAM, the second derivative revealed a minimum at 1642 cm^−1^, with two additional secondary minima at 1629 cm^−1^ and 1635 cm^−1^, all within the region associated with unordered protein structures. PNIPAM synthesized in water through free radical polymerization typically lacks significant tacticity [98], as confirmed by ^1^H NMR analysis showing the intensity of the peak at 1.42 ppm (see peak c in Figure 5) due to backbone methylene protons of racemic diads being nearly double those of the peak at 1.54 ppm due to meso diads [98]. Therefore, an intensity ratio of 1:2 between the peaks at 1.54 and 1.42 ppm suggests a similar proportion of meso and racemo diads, indicating negligible isotactic or syndiotactic structure. The absence of stereoregularity in the PNIPAM backbone corresponds to the absence of ordered chain conformation in the solid state, as indicated by the position of the amide I band in the infrared spectrum. The presence of secondary minima in the second derivative may arise from regions with varying hydrogen bond distances, possibly due to adsorbed and bonded water, which are known to persist even after lyophilization [99].

Analysis of the amide I band in HYB-P by the second derivative method revealed a minimum at 1623 cm^−1^, absent in PNIPAM alone, attributed to the keratin component exhibiting a β-sheet structure. A second main minimum appeared at 1644 cm^−1^, with a less intense minimum at 1635 cm^−1^, mainly representing disordered PNIPAM and keratin.

The second derivative of the amide I band in the HYB-M spectrum closely resembled that of pure PNIPAM, with two additional shoulders detected at 1622 cm^−1^ and 1650 cm^−1^, corresponding to the minima of keratin. These data indicate that in HYB-M, keratin maintains the same secondary structure as when it is isolated.

### 2.3. Temperature and pH Responsivness of Hybrid Microgels

**Figure 6:**
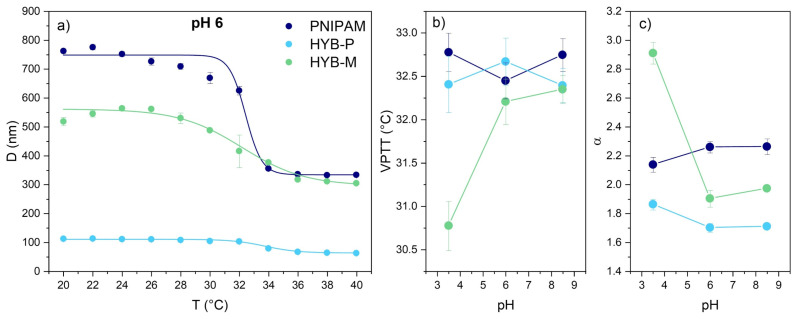
Data obtained by DLS analysis for PNIPAM, HYB-P and HYB-M: particle diameters as a function of temperature in the 20–40 °C range at pH 6 (**a**) and volume phase transition temperatures (VPTT) (**b**) and swelling ratios (α) (**c**) at pH 2.5, 6 and 8.5. Error bars for each point are standard deviations of three measurements; lines in (**a**) are fits of the data determined with a Boltzmann equation.

PNIPAM microgels are recognized for their negative response to temperature, collapsing upon heating [17]. The temperature at which this transition occurs is commonly referred to as the volume phase transition temperature (VPTT), which can be determined by measuring the hydrodynamic diameter of microgel particles at various temperatures (Figure 6a). In the absence of keratin, PNIPAM microgels were observed to collapse within the temperature range of 28–34 °C, reducing in diameter from 762 ± 3 nm at 20 °C to 333.3 ± 0.3 nm at 40 °C (Table 1). The VPTT, identified as the inflection temperature of the plot in Figure 6a [100], was measured to be 32.5 ± 0.2 °C at pH 6 and showed negligible changes when the pH was decreased to 3.5 or increased to 8.5 (Figure 6b). The ratio between the diameters in the swelled state (at 20 °C) and the collapsed state (at 40 °C), commonly referred to as the swelling ratio (α) [101], remained approximately 2.2 across all tested pH conditions. These properties and behaviors correspond to those previously reported for particles obtained under similar synthesis conditions, specifically in the absence of surfactant and with a 1.3% BIS/NIPAM molar ratio [32,33].

**Figure 7:**
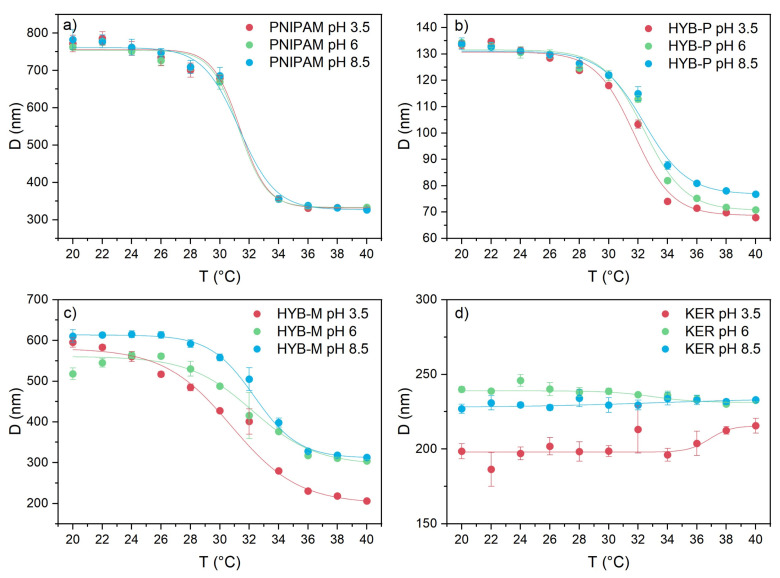
Particle diameters as a function of temperature in the 20–40 °C range at three different pH levels (3.5, 6 and 8.5) for PNIPAM (**a**), HYB-P (**b**), HYB-M (**c**) and KER (**d**). Error bars for each point are standard deviations of three measurements; lines are fits of the data determined with a Boltzmann equation.

As previously discussed, PNIPAM particles obtained in the presence of keratin (HYB-P) are significantly smaller than those of pure PNIPAM (Figure 6a), yet they still demonstrate temperature responsiveness, transitioning from a swollen state at room temperature to a collapsed state at 40 °C (Table 1). The diameters of the particles in the swollen state remained unaffected by the pH of the environment, while pH did influence the diameter in the collapsed state, with a slightly larger diameter observed at pH 8.5 (Figure 7). As also observed in PNIPAM–keratin hydrogels with a 10–20% of keratin content [54], the volume phase transition temperature (VPTT) showed minimal alteration due to both the incorporation of keratin during synthesis and variations in pH. Conversely, the swelling ratio was smaller than that of pure PNIPAM at all investigated pH values (Figure 6c), with a lesser decrease observed at acidic compared to neutral and basic pH values. This behavior suggests that the presence of the protein renders the system more rigid and hydrophobic, likely due to hydrogen bonding complexation. A similar reduction in swelling ability was previously reported for hybrid PNIPAM–keratin hydrogels at different crosslinking degrees [54] and for IPN microgels made of PNIPAM and poly(acrylic acid) [102,103].

The HYB-M sample, produced by simply mixing preformed PNIPAM microgels and the protein, maintained the temperature responsiveness of PNIPAM, with a VPTT comparable to that of pure PNIPAM at both pH 6 and 8.5 (Figure 6a). However, the VPTT was reduced by roughly 2 °C at pH 3.5 (Figure 6b). The microgels’ diameters were smaller than those of pure PNIPAM at any pH below the VPTT and only at pH 3.5 above the VPTT. The swelling ratio was intermediate between those of pure PNIPAM and HYB-P at pH 6 and 8.5 but was maximal at pH 3.5 (Figure 6c). This result can be attributed to the minimum value of the HYB-M diameter above the VPTT at pH 3.5. The reduced hydrophobicity of PNIPAM when complexed with the protein or decomplexation could account for this phenomenon. In the latter case, the protein would be free in solution. The hydrodynamic diameter of the protein in solution measured by DLS is approximately 250 nm (Figure 7)—smaller than that of PNIPAM—which may contribute to reducing the average value observed by DLS.

## 3. Conclusions

In this study, we successfully synthesized hybrid microgel particles of PNIPAM and keratin for the first time. Two distinct methods were employed: one involving the polymerization of NIPAM in the presence of keratin (HYB-P), and the other entailing the combination of a preformed PNIPAM microgel with keratin to form a physical mixture (HYB-M), resulting in hybrids with keratin contents of 20% and 25%, respectively. DLS and TEM analyses confirmed the formation of colloidal systems, with particles having diameters of approximately 110 nm for HYB-P and 518 nm for HYB-M. In the latter case, the addition of keratin led to a decrease in particle diameter compared to pristine PNIPAM microgel (approximately 750 nm), attributed to the absorption of the biopolymer on the microgel surface.

Distinct structures were observed for HYB-P and HYB-M: weak bond interactions between the two components of the hybrid material were confirmed for the latter, whereas a graft copolymer structure was suggested for the former. This finding was further supported by the ^1^H NMR spectra, where the complete absence of keratin signals only in HYB-P suggested strong coupling and/or entanglement with PNIPAM chains.

The temperature-responsiveness characteristic of PNIPAM microgels is retained in the hybrids, which have a VPTT very similar to the former (approximately 32.5 °C), indicating that the presence of keratin does not influence the transition temperature. However, it does impact the swelling ratio of the particles, as a reduction in swelling ability was observed for the hybrids, except for HYB-M at pH 3.5, where decomplexation of keratin from PNIPAM may occur.

In conclusion, these hybrids exhibit unique structural characteristics and responsiveness to temperature and even to pH variation, suggesting their potential for various biomedical applications.

## 4. Materials and Methods

### 4.1. Materials

N-isopropylacrylamide (NIPAM) (Sigma-Aldrich, St. Louis, MO, USA, purity 97%) was recrystallized from hexane. N,N′-methylene-bis-acrylamide (BIS) (Sigma-Aldrich, purity ≥99.5%) was recrystallized from methanol, and the crystals were washed with cold diethyl ether before being dried. Sodium dodecyl sulfate (SDS) (Sigma-Aldrich, purity 98%), potassium persulfate (KPS) (Fluka, Charlotte, NC, USA, purity 98%), L-cysteine (Carlo Erba, Emmendingen, Germany, RPE) and 5,5′-dithiobis-(2-nitrobenzoic acid) (DTNB) (Sigma Aldrich, purity ≥98%—a bioreagent suitable for the determination of sulfhydryl groups) were used as received. High-molecular-weight keratin (Ker) powder (≈50 kDa) extracted from raw wool was kindly donated by Kerline Srl (Bologna, Italy); elemental analysis: C = 47.2 ± 0.6%, N = 10.4 ± 0.6%, H = 7.1 ± 0.2%, S = 5.20 ± 0.04%. It was solubilized in ultrapure water at ~1 wt.% concentration by sonication and then stirred overnight at room temperature. Ultrapure water was obtained using a Sartortius Arium Mini water purification system fed with pretreated deionized water. A dialysis membrane, SpectraPor^®^ 1, MWCO 6–8 kDa (Spectrum Laboratories, Inc., Piscataway, NJ, USA), was soaked in distilled water for 2 h and then thoroughly rinsed before use. Saline–sodium phosphate-EDTA buffer was obtained by solubilization of pHast Pack™ (Sigma Aldrich) in ultrapure water. All other solvents (Carlo Erba, RP grade) were used as received.

### 4.2. Microgel Synthesis

*PNIPAM*: 1.1663 g of NIPAM and 0.021 g of BIS were solubilized in 90 mL of ultrapure water. The solution was poured into a 250 mL jacket reactor, deoxygenated by bubbling nitrogen for 1 h, heated at 70 ± 1 °C and then 0.068 g of KPS (dissolved in 10 mL of deoxygenated water) was added to initiate the polymerization. The reaction was carried out for 4 h under a nitrogen atmosphere at a constant mixing speed of 200 rpm. The obtained dispersion was left to cool at room temperature and then was purified by dialysis against distilled water with frequent water change for 2 weeks. The concentration was 1.00 ± 0.05 wt.%. Elemental analysis: C = 56.7 ± 0.5%, N = 11.8 ± 0.1%, H = 9.37 ± 0.04%, S = 0%.

*HYB-P*: 1.164 g of NIPAM and 0.021 g of BIS were solubilized in 61.3 mL of ultrapure water and then mixed with 38.78 g of keratin solution (0.99 ± 0.02 wt.% in water) in a 250 mL jacket reactor. The mixture was deoxygenated by bubbling nitrogen for 1 h, heated at 70 ± 1 °C and then 0.070 g of KPS (dissolved in 10 mL of deoxygenated water) was added to initiate the polymerization. The reaction was carried out for 4 h under a nitrogen atmosphere at a constant mixing speed of 200 rpm. The obtained dispersion was left to cool at room temperature and then was purified by dialysis against distilled water with frequent water change for 2 weeks. The concentration was 1.05 ± 0.04 wt.%. Elemental analysis: C = 56.48 ± 0.09%, N = 11.70 ± 0.06%, H = 8.81 ± 0.08%, S = 0.99 ± 0.01%.

*HYP-M* was prepared by mixing 34.76 mL of PNIPAM dispersion (C_w_: 1.00 ± 0.05 wt.%) and 12.00 mL of keratin solution (0.99 wt.%). Elemental analysis: C = 54.3 ± 0.5%, N = 10.9 ± 0.2%, H = 8.7 ± 0.1%, S = 1.20 ± 0.04%.

### 4.3. Characterization Instruments and Methods

Solid contents of dispersions were measured by comparing the original weight of a 150 mg sample with the same sample after heating for 2 h at 150 °C in an oven.

^1^H NMR spectra were registered on a Varian Mercury Plus 400 MHz (Varian Inc., Palo Alto, CA, USA) spectrometer equipped with autosampler in D_2_O at room temperature. Sample concentration was approximately 15 g/L. Chemical shifts were referred to TMS as an external standard.

Infrared spectroscopy analyses were accomplished with a PerkinElmer (Perkin-Elmer Inc., Norwalk, CT, USA) Spectrum 100 FT-IR spectrometer equipped with a Universal ATR accessory. SpectraGryph 1.2.16.1 software was used for spectral analyses [24].

Dynamic light scattering (DLS) measurements were carried out using a Zetasizer Nano S90 (Malvern Instruments, Malvern, UK) equipped with a HeNe laser (633 nm, 5 mW) and a Peltier Cell for temperature control. Samples were diluted to approximately 0.1 mg/mL with ultrapure water before analysis. Measurements were carried out in triplicate in the 25–40 °C temperature range at 2 °C intervals, and an average result was generated using Zetasizer 8.02 software, with the related errors calculated as standard deviations. Intensity size distributions were generated by the same software by the CONTIN method. 

Transmission electron microscopy (TEM) analysis was accomplished with a Talos™ L120C transmission electron microscope (Thermo Fisher Scientific Inc. Waltham, MA, USA) equipped with a LaB_6_ thermionic source (120 kV, 5 μA). Samples for observation were prepared by dropping dispersions (0.001 wt.% concentration) on copper grids covered with a thin carbon film (Merck, Darmstadt, Germany, CF200-Cu-50, 200 mesh, film thickness 5–6 nm) and leaving them to dry for ~30 min.

CHNS/O analysis was accomplished by using a FLASH2000 instrument (ThermoFisher Scientific, Waltham, MA, USA). Two-milligram freeze-dried samples were analyzed in triplicate, and the average values were used as data.

### 4.4. Ellman Assay

A quantity of 250 μL of sample (~1 wt.% concentration) was diluted with 2.5 mL of buffer and 50 μL of TNB solution (4 g/L in water) was added. The sample was incubated for 15 min at room temperature and then analyzed in the 300–600 nm spectral range using a Cary 100 Scan UV-Vis spectrophotometer (Agilent Technologies, Santa Clara, CA, USA). The difference between the absorbance values at 412 nm of the sample and a reference obtained without the DTNB solution (Ellman reagent) was used for quantification purposes by exploiting a calibration plot obtained with L-cysteine solutions.

## Figures and Tables

**Table 1:** Particles’ diameters (D) at 20 °C and 40 °C determined by DLS analysis, polydispersity indices (PDIs), temperatures of volume phase transition (VPTT), swelling ratios (α) and sulfur contents determined by elemental analysis.

Sample	D_20°C_ (nm)	D_40°C_ (nm)	PDI	VPTT (°C)	α ^1^	%S
PNIPAM	762 ± 3	333.3 ± 0.3	0.043	32.5 ± 0.2	2.29 ± 0.01	0
HYB-P	111 ± 2	62.0 ± 0.4	0.207	33.8 ± 0.5	1.80 ± 0.04	0.99 ± 0.01
HYB-M	518 ± 5	304.1 ± 0.9	0.153	32.2 ± 0.3	1.70 ± 0.02	1.20 ± 0.04
KER	240.0 ± 0.6	232.7 ± 0.4	0.185	-	1.031 ± 0.003	5.20 ± 0.04

^1^ D_40°C_/D_20°C_.

## Data Availability

The original contributions presented in the study are included in the article/supplementary materials; further inquiries can be directed to the corresponding authors.

## Supplementary Materials

The following supporting information can be downloaded at: https://www.mdpi.com/article/10.3390/gels10060411/s1, Figure S1: ATR spectrum of PNIPAM; Figure S2: ATR spectrum of HYB-P; Figure S3: ATR spectrum of HYB-M; Figure S4: ATR spectrum of KER; Figure S5: 1H-NMR spectrum of PNIPAM in D_2_O; Figure S6: 1H-NMR spectrum of HYB-P in D_2_O; Figure S7: 1H-NMR spectrum of HYB-M in D_2_O; Figure S8: 1H-NMR spectrum of KER in D_2_O.
