# Supplementary material for: Keratin–PNIPAM Hybrid Microgels: Preparation, Morphology and Swelling Properties
Source: Gels. 2024 Jun 20;10(6):411. doi: 10.3390/gels10060411 (PMC11202486; doi:10.3390/gels10060411)
Supplement: Supplementary file 1 [file gels-10-00411-s001.zip › Supplementary Materials.pdf]

Supplementary Materials of: "Keratin/PNIPAM hybrid microgels: preparation, morphology and swelling properties"

Elena Buratti[*1], Maddalena Sguizzato[1], Giovanna Sotgiu[2], Roberto Zamboni[2], and Monica Bertoldo[1]*

[1]*Department of Chemical, Pharmaceutical and Agricultural Sciences, University of Ferrara, via. L. Borsari, 46, 44121, Ferrara, Italy; e-mail@e-mail.com*

[2] *Institute for Organic Synthesis and Photoreactivity (ISOF), National Research Council, Via Gobetti 101, Bologna, 40129, Italy*

**Correspondence: elena.buratti@unife.it*

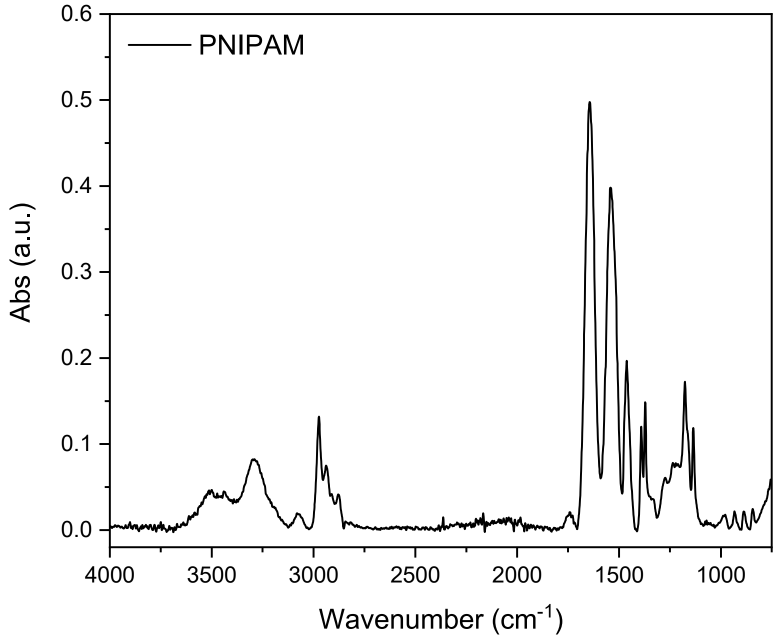

Figure S1. ATR spectrum of PNIPAM

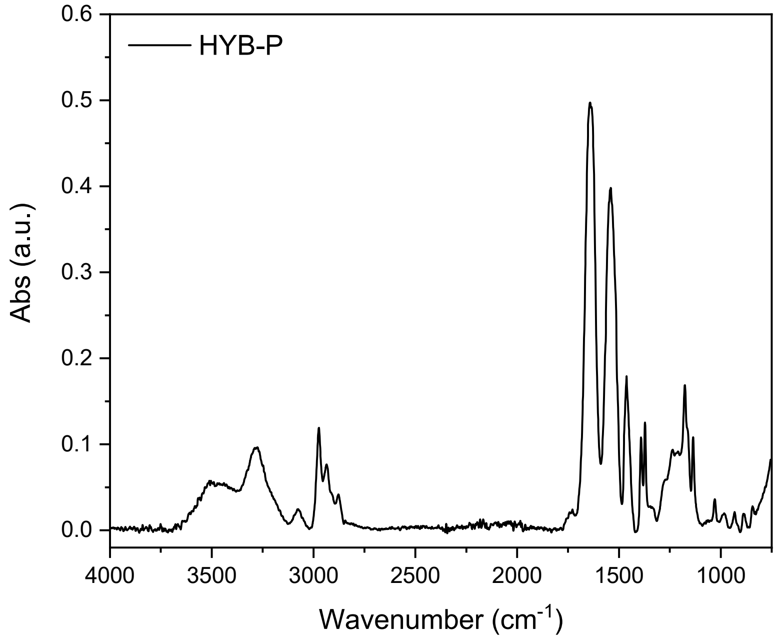

Figure S2. ATR spectrum of HYB-P

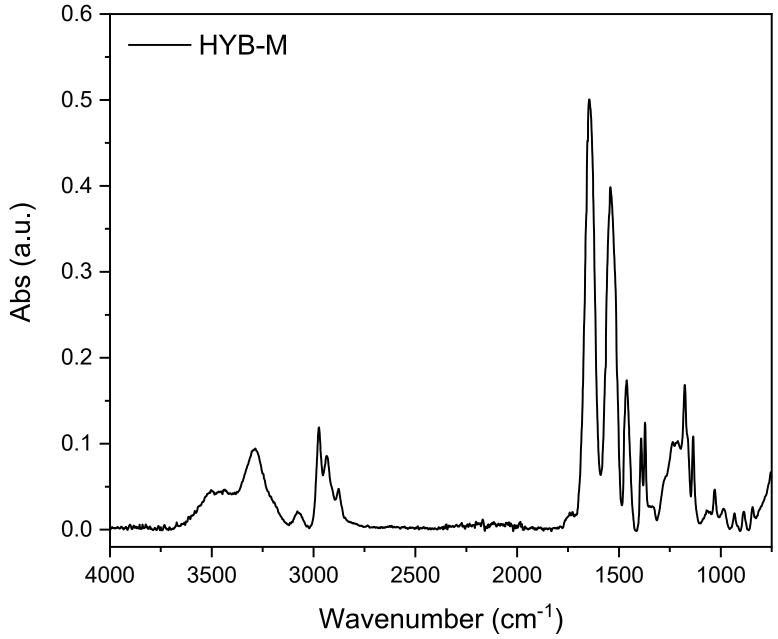

Figure S3. ATR spectrum of HYB-M

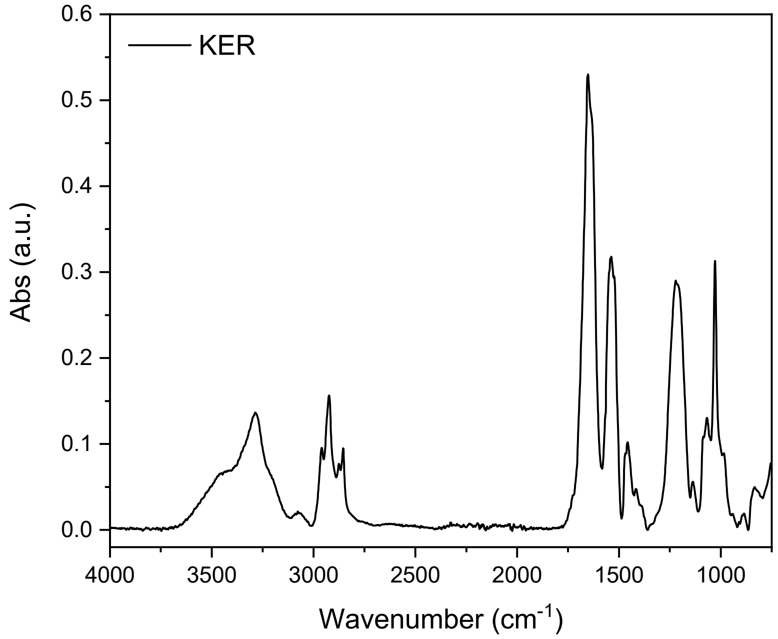

Figure S4. ATR spectrum of KER

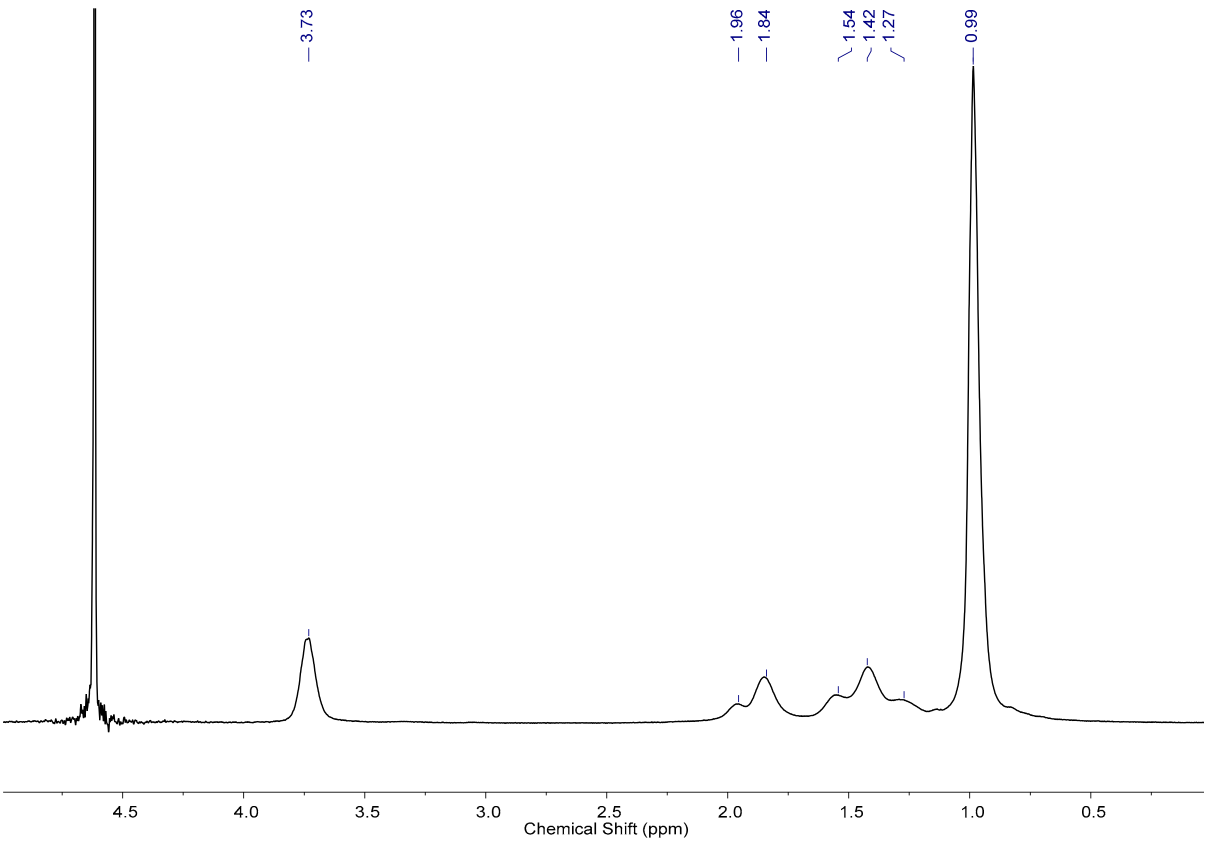

Figure S5. ^{1}H-NMR spectrum of PNIPAM in D_2O

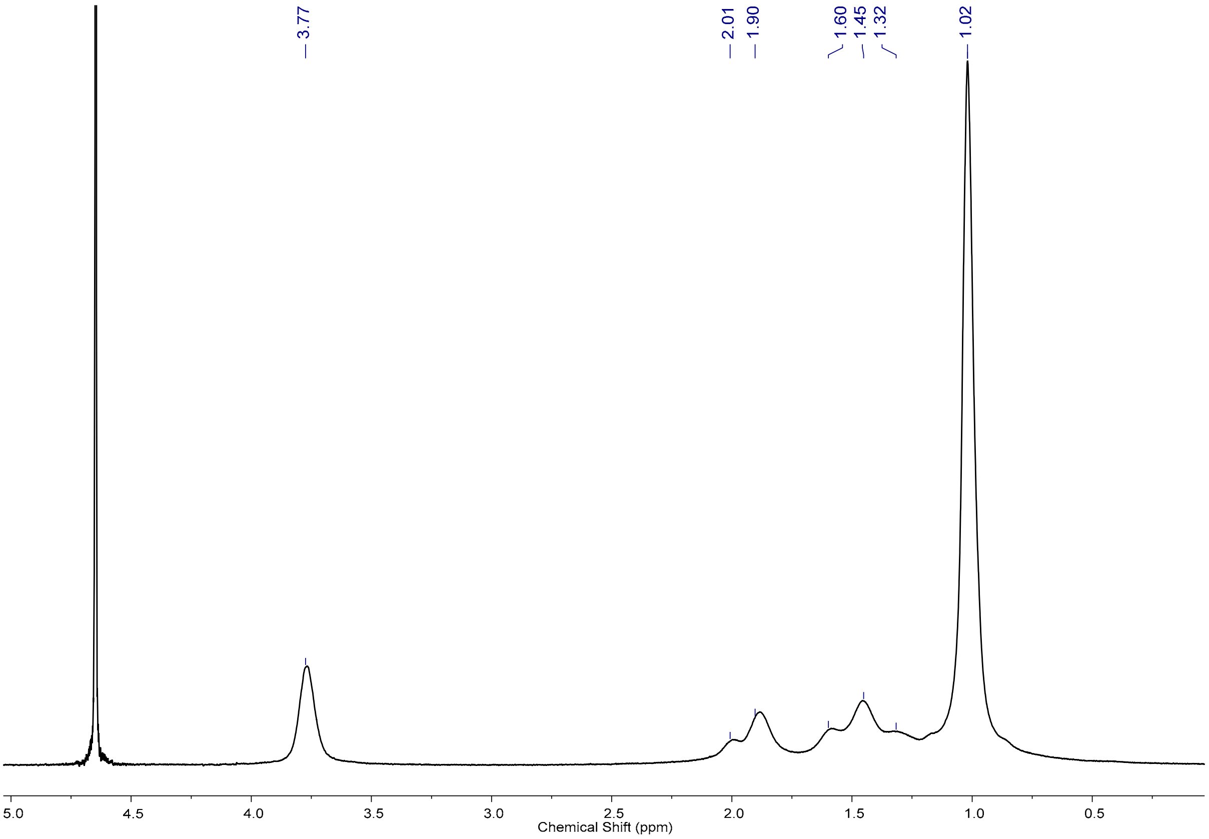

Figure S6. ^{1}H-NMR spectrum of HYB-P in D_2O

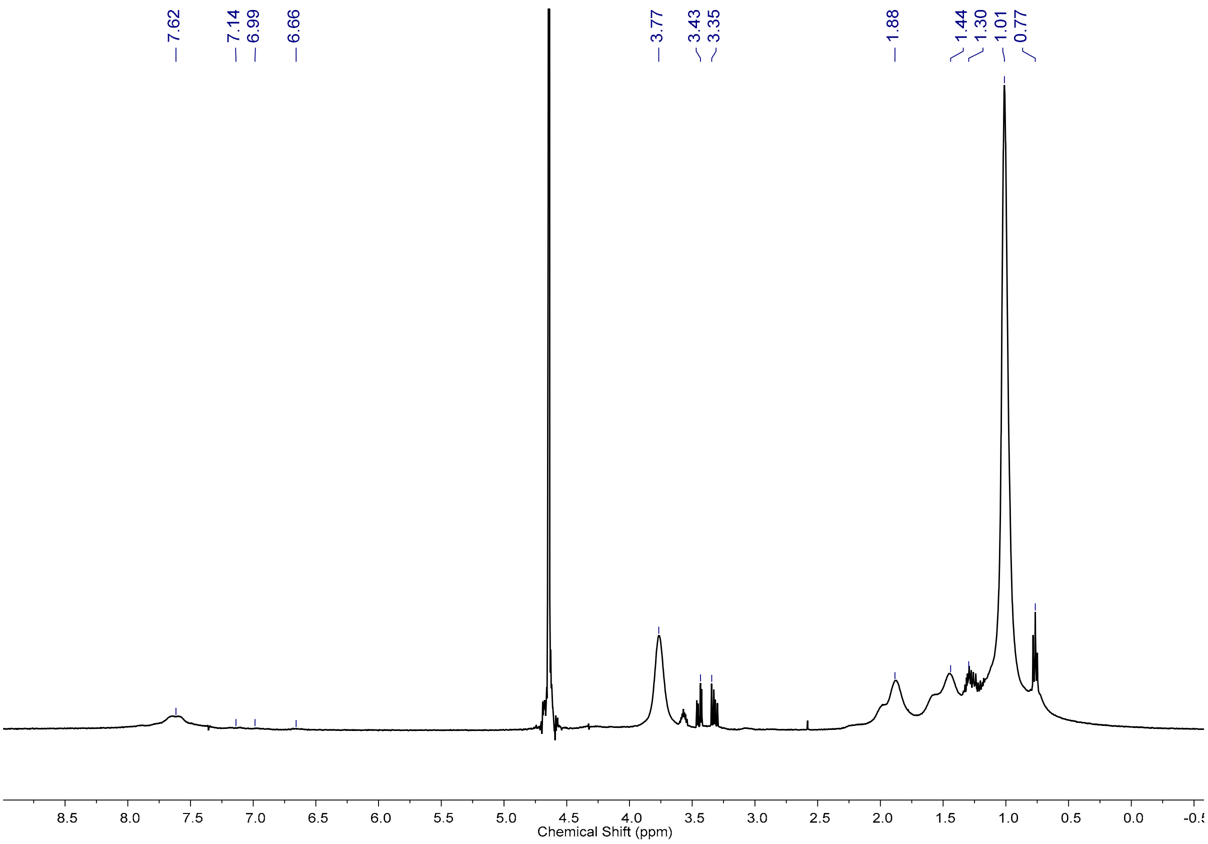

Figure S7. ^{1}H-NMR spectrum of HYB-M in D_2O

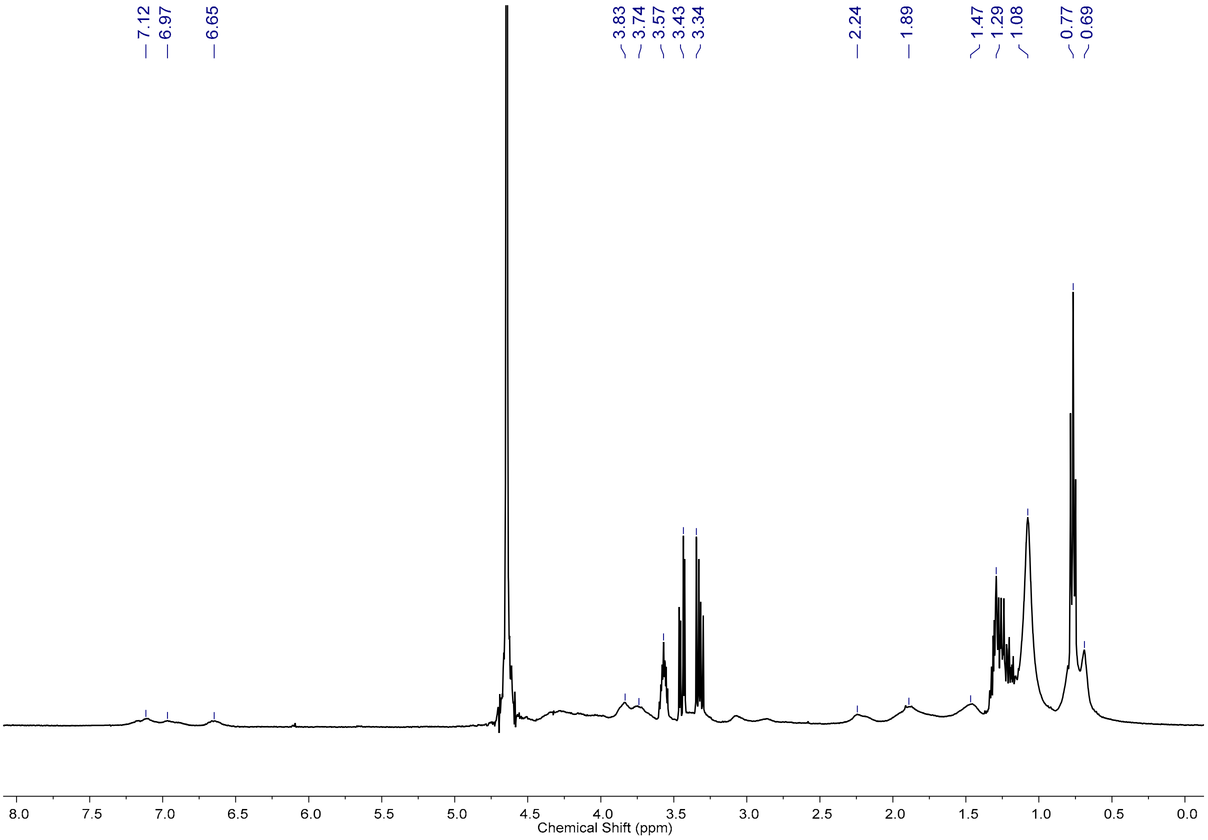

Figure S8. ^{1}H-NMR spectrum of KER in D_2O
